# Staurosporine Induces the Generation of Polyploid Giant Cancer Cells in Non-Small-Cell Lung Carcinoma A549 Cells

**DOI:** 10.1155/2018/1754085

**Published:** 2018-10-10

**Authors:** Alexander Glassmann, Carmen Carrillo Garcia, Viktor Janzen, Dominik Kraus, Nadine Veit, Jochen Winter, Rainer Probstmeier

**Affiliations:** ^1^Life Science Inkubator, Bonn, Germany; ^2^Department of Internal Medicine III, Division of Hematology and Oncology, University of Bonn, Bonn, Germany; ^3^Department of Prosthodontics, Preclinical Education, and Material Science, University of Bonn, Bonn, Germany; ^4^Neuro- and Tumor Cell Biology Group, Department of Nuclear Medicine, University of Bonn, Bonn, Germany; ^5^Oral Cell Biology Group, Department of Periodontology, Operative and Preventive Dentistry, University of Bonn, Bonn, Germany

## Abstract

Cultivation of A549 non-small-cell lung carcinoma (NSCLC) cells in the presence of staurosporine (SSP) leads to a reduction or a lack of proliferation in a concentration-dependent manner. This inhibition of proliferation is accompanied by the generation of polyploid giant cancer cells (PGCCs) that are characterized by cell flattening, increased cell size, polyploidy, and polynucleation as determined by crystal violet staining, BrdU and DiI labelling, and flow cytometry as well as video time-lapse analysis. Continuous SSP treatment of A549 cells can preserve PGCCs for at least two months in a resting state. Upon removal of SSP, A549 PGCCs restart to divide and exhibit a proliferation pattern and cellular morphology indistinguishable from cells where PGCCs originally derived from. Thus, SSP-treated A549 cells represent a simple and reliable experimental model for the reversible generation of PGCCs and their subsequent experimental analysis.

## 1. Introduction

Tumor cells often acquire resistance towards insults that otherwise would interfere with the proliferation and or survival potential of these cells. Such a resistance against antiproliferative therapies can be generated, for example, via the expression of multidrug resistance pumps or a reduced cycling rate. In particular, tumor stem cells are inter alia characterized by long-term self-renewal, a low proliferation rate, and resistance towards anticancer drugs and irradiation [1, 2]. Another intriguing possibility that leads to a reduced proliferation rate is the occurrence of reversible tumor cell senescence or the generation of polyploid giant cancer cells (PGCCs). Such cells that are characterized by multinucleation and cell cycle arrest were first characterized almost two decades ago [3, 4]. As it is the case for tumor stem cells, these cells are resistant towards drugs that interfere with tumor cell proliferation, such as DNA-damaging drugs. Moreover, PGCCs have been demonstrated to possess stem cell-like properties, as they form spheroids in vitro and generate tumors in mice [5, 6]. Recently, it has also been demonstrated that PGCCs could function as blastomere-like stem cells [7]. Thus, PGCCs may play fundamental roles in tumor heterogeneity, stemness, and resistance [6].

The relationship between PGCCs and senescent cells is still a matter of discussion, whereby also a standardized nomenclature is missing. PGCCs have been described as nondividing flattened tumor cells that are irreversibly arrested either in the G0/G1 or G2/M state and express *β*-galactosidase activity [8, 9]. On the contrary, PGCCs have also been characterized as not senescent due to the lack of *β*-galactosidase staining [10]. Moreover, subpopulations of cancer cells that have been described to be in a state called “pseudosenescence” possess the potential to restart proliferation and, in consequence, are able to repeatedly initiate cancer [11].

To our knowledge, the number of easy handling protocols that describe the generation and maintenance of PGCCs in high yields is restricted. The enrichment of PGCCs that are already present as a minor subpopulation in cultured ovarian cancer cell lines as well as primary cancer has been reported. CoCl_2_ treatment of such cultures, which mimics hypoxic conditions, led to the death of normal cancer cells, whereas giant cells remained alive [10]. In colon cancer cells, CoCl_2_ treatment leads to the generation of PGCCs with characteristics of stem cells [12].

The small kinase inhibitor staurosporine (SSP) is an alkaloid derived from the bacterium *Streptomyces stauroporeus*. The molecule is not of clinical interest due to its broad inhibition profile [13]. In a detailed study, SSP has been shown to interact with most of the kinases representing the human kinome [14]. On the cellular level, SSP interferes with cell migration, proliferation, differentiation, and survival in a multifaceted manner [15, 16]. Also, we have recently shown that SSP mediates the conversion of small cell lung carcinoma cells into a neuron-like process-bearing phenotype [17], whereby the broad pattern of SSP-induced effects is more restricted with different SSP analogs that exhibit a higher substrate specificity [18].

Here, we describe that continuous treatment with SSP provides a simple procedure for the generation and maintenance of large amounts of reversibly growth-arrested PGCC non-small-cell lung carcinoma (NSCLC) A549 cells.

## 2. Materials and Methods

### 2.1. Cell Lines and Culture Conditions

NSCLC A549 cells were maintained in DMEM 10% fetal calf serum (FCS).

### 2.2. Cell Proliferation and Viability Assay

To determine cell viability and proliferation, crystal violet and LDH assays were performed: For the crystal violet assay, cells were seeded in 96 well plates, incubated overnight in normal culture medium, and then further cultured in DMEM containing 1% FCS in the absence or presence of experimental compounds. Cells were then fixed with formaldehyde, stained for 1 h with 0.05% crystal violet in Aqua dest., washed, and air-dried. 150 *μ*L of methanol was added per well, and the optical density at 540 nm was measured. The LDH assay was carried out as described previously with the “LDH Cytotoxicity Assay Kit” from Roche [19].

### 2.3. BrdU Labelling

For bromodeoxyuridine (BrdU) labelling, adherent A549 cells were incubated with 10 *μ*M BrdU (Sigma) in DMEM 10% FCS for 4 h at 37°C in a CO_2_ incubator. Cells were then washed three times with phosphate-buffered saline (PBS) and fixed for 10 min in PBS containing 4% formaldehyde at room temperature (RT). Cells were washed twice in PBS and incubated in 4N HCl containing 1% Triton X-100 for 10 min at RT. After washing with PBS, cells were blocked with 2% bovine serum albumin (BSA) in PBS for 10 min. Cells were then incubated with anti-BrdU antibodies (Sigma) diluted in PBS, 0.2% BSA for 30 min at RT, washed with PBS, incubated with anti-mouse IgG-Cy3 (Sigma) for 30 min at RT in the dark, washed again, and embedded for fluorescence microscopic analysis.

### 2.4. DiI Labelling

For labelling with Vybrant DiI (Thermo Fisher), suspensions of A549 cells (1 × 10^6^ cells per 5 mL in serum-free DMEM) were incubated with DiI at a final concentration of 10 *μ*M for 30 min at 37°C. Cells were then washed three times in DMEM, finally seeded into Petri dishes in DMEM 10% FCS, and allowed to adhere overnight before compounds were applied.

### 2.5. Flow Cytometric Analysis

Flow cytometry was performed on a BD FACSCanto II instrument, and the data were analyzed with the FlowJo software. Cell cycle status was analyzed as already described [17, 18]. Briefly, A549 cells were trypsinized, collected in PBS, fixed and permeabilised following BD BrdU Flow kit manufacturer's instructions, incubated with anti-human Ki67 FITC conjugated antibody (BD Pharmingen), and counterstained with DAPI to exclude debris and necrotic cells.

### 2.6. Video Time-Lapse Analysis

Cells were applied to 24-well plates and allowed to adhere overnight. One hour prior to time-lapse analysis, SSP was added to a final concentration of 50 nM, and analysis was carried out as described [20]. Briefly, plates were transferred to a heated (37°C), gassed (5% CO_2_/air), and humidified chamber fitted onto an inverted microscope (Leica DM IRE2 HC Fluo) with a motorized cross-stage. Images were recorded every 10 min for 24 hours.

### 2.7. Cell Size Analysis

A549 cells were cultivated in DMEM 10% (FCS) in the absence or presence of 50 nM SSP. Photomicrographs were taken, and maximal cell diameters were determined with the help of ImageJ (http://www.imagescience.org/).

## 3. Results

### 3.1. Staurosporine Inhibits Proliferation of A549 Cells

**Figure 1:**
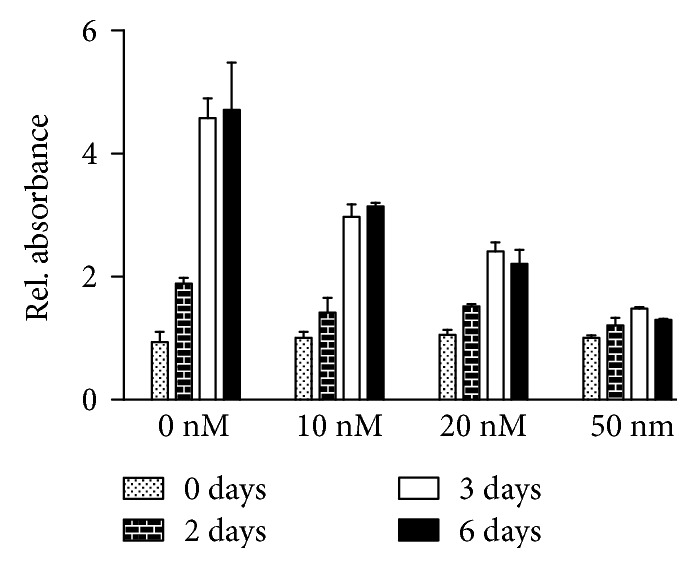
Proliferation of A549 cells in the presence of SSP. A549 cells were cultivated with the indicated concentrations of SSP for 2, 3, or 6 days in 96-well plates. Cells were stained with crystal violet as specified in Materials and Methods. Absorbance of cells at day 0 was set at 1.0, and the other values were adjusted accordingly. Columns represent mean values + standard deviation (SD).

A549 cells that were cultivated for one to six days in the presence of staurosporine with different concentrations showed a concentration-dependent decrease in their proliferative activity. Different batches of A549 cells varied in their sensitivity towards SSP-induced growth inhibition, but in general, an almost complete inhibition was observed in the presence of 50 nM SSP. A typical experiment is shown in Figure 1, where cells were incubated for up to 6 days. As in this type of experiments, no change of the culture medium was performed; the proliferation rate decreased after longer time periods also when no or low SSP concentrations were present. To rule out dominant cytotoxic effects, lactodehydrogenase (LDH) assays were performed. When cells were treated for 24 h with up to 200 nM SSP, no cytotoxic effects were observed.

### 3.2. Staurosporine Induces PGCC Formation

**Figure 2:**
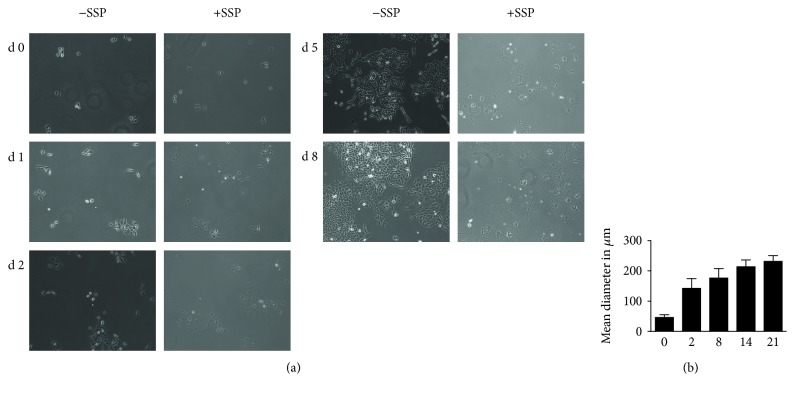
Micrographs (a) and size analysis (b) of A549 cells cultivated in the absence or presence of SSP. A549 cells were seeded at low density in Petri dishes and treated for up to 8 (a) or 21 (b) days without or with 50 nM SSP. In (b), at least 20 cells were analyzed for each time point. d: day(s).

To analyze morphological changes, A549 cells were initially seeded at low density and cultivated in the absence or presence of 50 nM SSP for up to 21 days. Microscopic analysis of such cultures revealed a continuous proliferation of tumor cells in the absence of SSP that led to the formation of large islands of confluent cell layers after 4 days. Untreated A549 cells with a mean diameter of about 45 *μ*m exhibited rounded to spheroid shapes and harboured small processes when present as isolated cells (Figure 2(a)). In contrast, SSP-treated A549 cells exhibited a considerable cell flattening already 2 h after drug addition. This increase in cell surface area was more pronounced during the first two days but continued at a lower extent up to 21 days (the latest time point analyzed, Figure 2(b)). Cells that exhibited an at least threefold larger diameter compared to untreated cells were defined as PGCCs. According to this criterion, after a SSP treatment of 2 days, 50% of A549 cells could be designated as PGCCs, and after longer treatments, that is, 8, 14, and 21 days, almost without exception, 100% of A549 cells could be designated as PGCCs. After 21 days of SSP treatment, A549 cells showed a mean diameter of 230 *μ*m, that is, about 5-fold the mean diameter of untreated cells (Figure 2(b)). Occasionally, also cells with diameters of 400 to more than 500 *μ*m were observed. In such cell populations, cell divisions were almost absent, as revealed inter alia by video time-lapse analysis.

**Figure 3:**
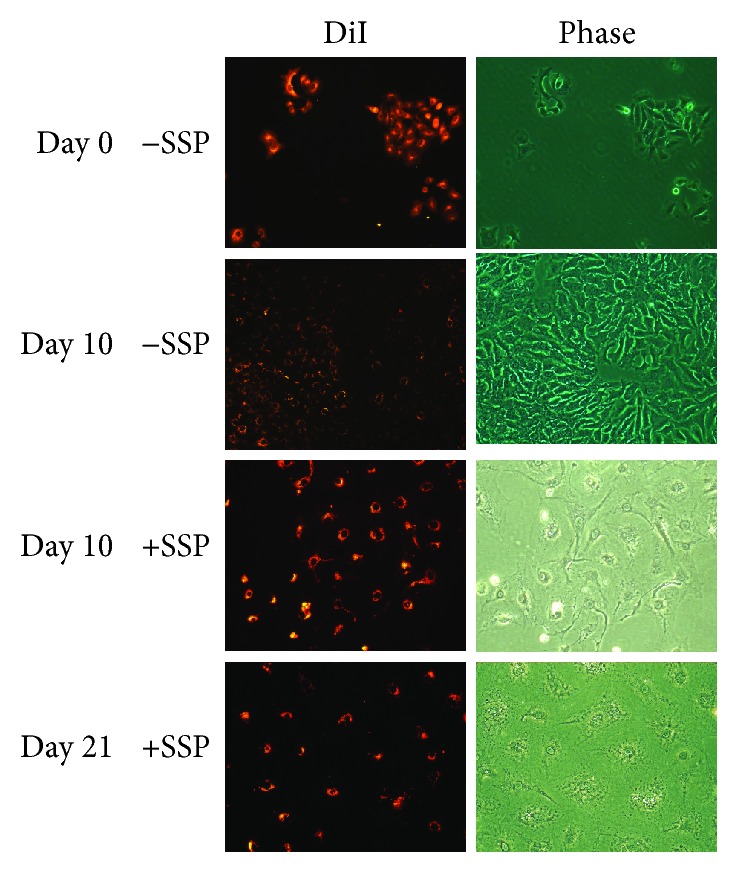
Micrographs of DiI-labelled A549 cells cultivated in the absence or presence of SSP. A549 cells were labelled with DiI, seeded at low density in Petri dishes, and treated for up to 21 days without or with 50 nM SSP. Fluorescence and corresponding phase micrographs are shown.

**Figure 4:**
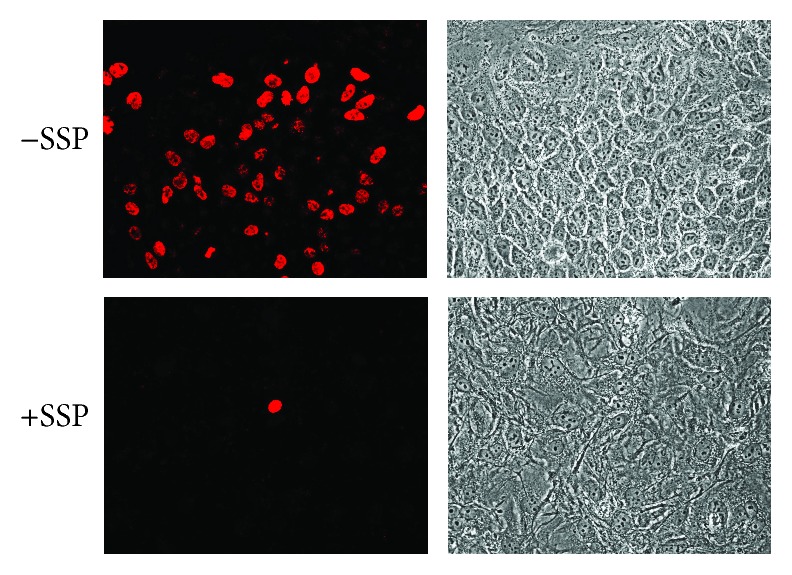
SSP-dependent BrdU incorporation in A549 cells. Cells were seeded in 24-well plates at high density and cultivated for further 3 days in the absence or presence of 50 nM SSP. Cells were then pulsed for 4 h with BrdU. BrdU incorporation was visualized with anti-BrdU antibodies and secondary fluorescently labelled antibodies. Fluorescence and corresponding phase micrographs are shown.

To further underline the absence of cell divisions, A549 cells were either labelled with DiI (Figure 3) or BrdU (Figure 4). In untreated cells, DiI labelling was diminished in single cells with time in culture due to extensive cell proliferation, whereas it became more conserved in nonproliferative SSP-treated PGCCs (Figure 3). It has to be noted that the label becomes less uniformly distributed with time in culture, a phenomenon that is treatment independent. Also, labelling experiments with BrdU revealed an almost abolished proliferation in SSP-treated A549 cells (Figure 4). The absence of proliferation and the fast generation of flattened PGCCs upon SSP treatment could also be documented in video time-lapse analysis. We also tried to verify *β*-galactosidase activity in SSP-treated A549 cells but could detect only a residual activity, which could not be substantially visualized in photomicrographs.

### 3.3. SSP Induces the Formation of Polyploid and Polynucleated A549 Cells

**Figure 5:**
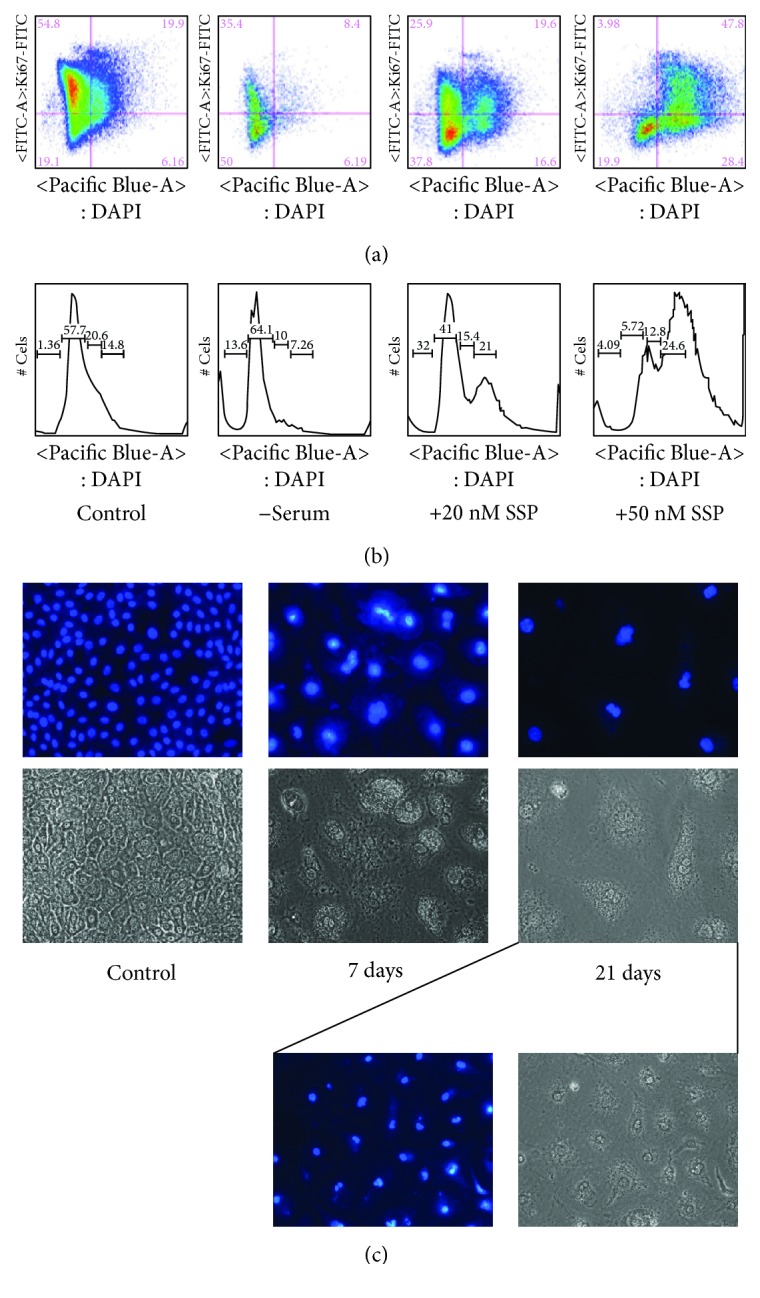
SSP induces polyploidy and polynucleation in A549 cells. (a, b) FACS analysis of A549 cells that had been cultivated in normal growth medium with (control) or without (−serum) 10% FCS or in growth medium with 10% FCS containing 20 or 50 nM SSP. FACS plots are shown in (a), whereas in (b), the respective relative cell numbers in representative sections of micrographs in (a) are given on the *y*-axis. (c) DAPI staining to reveal the nuclear morphology of A549 cells that had been cultivated in the absence or presence of 50 nM SSP for 7 or 21 days. The corresponding phase micrographs are also given. In addition, a lower magnification of 21-day treated A549 cells is shown at the right bottom of (c) to reveal a larger cell number.

To further analyze the growth characteristics of SSP-treated A549 cells, we performed FACS analyses (Figure 5). When A549 cells were serum-starved for 32 hours, the expected accumulation of cells in G0/G1 phase was observed in comparison to cells that were cultivated in the presence of 10% FCS (Figures 5(a) and 5(b)). Treatment of A549 cells for 32 hours with 20 and more pronounced with 50 nM SSP led to an accumulation of cells in the G2/M phase (Figures 5(a) and 5(b)), without signs of apoptotic cell death as revealed by annexin V staining (not shown). Predominantly, the FACS profile of cells treated with 50 nM SSP hints the presence of polyploidal effects, indicated by the pronounced displacement of the profile to the right portions of the diagrams (Figures 5(a) and 5(b)). Micrographs of DAPI-stained A549 cells revealed the presence of a significant fraction of polynucleated PGCCs in SSP-treated but not in untreated cultures (Figure 5(c)), whereby polynucleated PGCCs became more pronounced after longer culture periods.

### 3.4. SSP-Induced Inhibition of Proliferation in PGCC A549 Cells Is Reversible

**Figure 6:**
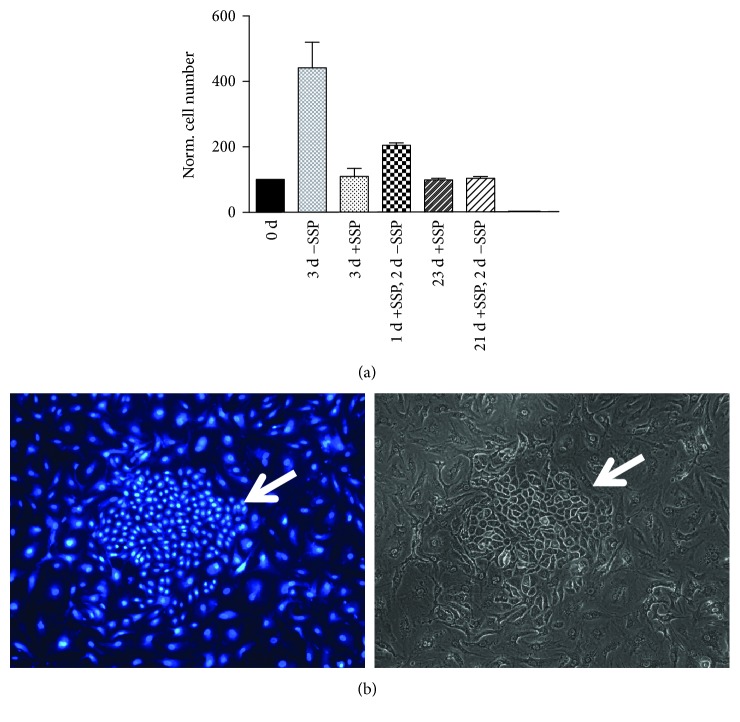
SSP-induced stop of proliferation is reversible. (a) Short- and long-time cultures of A549 cells in the absence or presence of 50 nM SSP. A549 cells were cultivated at the indicated time points with or without 50 nM SSP, stained with DAPI, and the cell number in selected areas was determined microscopically. Cell number at day zero (d 0) was set as 100%, and the other values were adjusted accordingly. (b) A549 cells that had been cultured in the presence of 50 nM SSP for three weeks were cultured in the absence of SSP for further three weeks. A colony of dividing cells in a dawn of still more flattened cells is indicated with an arrow. Fluorescence (DAPI label) and corresponding phase micrographs are shown.

A549 cells that had been treated with SSP and generated PGCCs restarted to proliferate upon SSP removal. However, the kinetics differed dependent on the duration of the prevenient SSP incubation. In A549 cells that had been treated with SSP for 24 h, proliferation restarted within the subsequent 48 h (Figure 6(a)). In contrast, in A549 cells that had been cultured in SSP-containing medium for three weeks, profound reproliferation started about two to three weeks after SSP removal, and often, fast proliferating cell clones became visible that were located within a layer of still partially flattened cells (Figures 6(a) and 6(b)). However, detailed video time-lapse analysis revealed that some cells (≤3%) were dividing already 48 h after SSP removal.

## 4. Discussion

It has become clear during the last years that PGCCs are involved in fundamental processes during tumor formation and progress. Thus, it seems necessary that this subpopulation of cancer cells gains more attention in the different aspects of cancer biology and therapy. For such a purpose, simple model systems are needed that allow the detailed investigation of such cells. In the present study, we have demonstrated that SSP-treated A549 cells may serve as such a model system. As SSP-treated A549 PGCCs can be subcultured and yet retain their PGCC phenotype, they can be easily handled if the well format of plates needs to be changed caused by experimental reasons. Moreover, depletion of SSP allows the regeneration of dividing cells that can be transformed again into PGCCs upon another continuous addition of SSP.

Our study did not allow identifying the precise mechanism on how PGCCs regain their proliferative activity upon SSP depletion and, in addition, if all PGCCs are able to do so. A549 cells contain a side population, comprising 24% of the total cell population that possess stem cell properties [21]. As this subpopulation overexpresses ABC transporters, it may be possible that such cells differ in their response raised by SSP removal in comparison to nonside population A549 cells. For PGCCs, unusual asymmetric cell divisions have been proposed that allow depolyploidization to varying degrees [6, 10]. We have observed by FACS analysis that in SSP-depleted cultures of A549 cells the amount of polyploidy decreases, but the underlying cellular mechanisms remain to be elucidated. It would also be of interest to evaluate if during depolyploidization heterogeneous cellular subclones can develop that differ in therapy-relevant properties, such as sensitivity towards drugs or irradiation [22].

At high concentrations, SSP is an almost general inducer of apoptosis, irrespective of the cell type, as it has been shown to provoke cell death in a number of organ cultures in egg cells as well as in blastomeres, even when protein synthesis is inhibited [23]. At lower concentrations, it leads to cell cycle arrest, often accompanied with alterations in the cell state. These changes are dependent on cell density, SSP concentration, and the kinetics of SSP application. In A549 cells, for example, it has been demonstrated that incubation with SSP (i) at concentrations of 1 to 100 nM for maximal 48 h leads to a reduction in proliferation cell cycle arrest in G2/M cell rounding and signs of apoptosis at 100 nM SSP [24] but (ii) at a concentration of 5 nM for up to 4 days leads to cell cycle arrest in G0/G1, whereby considerable cytotoxic effects were only observed at SSP concentrations of 10 *μ*M [25]. These data could indicate a different impact of SSP on specific kinases when used at various concentrations and for variable time periods [14]. In preliminary experiments, we have found that in the continuous presence of SSP, some glioma cell lines differentiate into PGCCs, although the majority of cell lines only expresses a highly branched cell morphology with neurite-like processes, as previously reported by us for SCLC and PC12 cells [17, 26].

## 5. Conclusion

PGCCs are a relevant target to be addressed in the context of cancer therapy. However, the methodical repertoire to generate and cultivate such cells is limited. The SSP-mediated generation of PGCCs in the A549 tumor cell line we have documented here presents a simple approach that allows the rapid generation of PGCCs at high yields in a reversible manner and thus permits a detailed analysis of desired parameters in various well formats. However, it remains to be determined if this approach can be transferred to a representative number of other cell lines derived from other major human tumor entities.

## Data Availability

The data used to support the findings of this study are available from the corresponding author upon request.
